# Polymeric Nanoparticles for Increasing Oral Bioavailability of Curcumin

**DOI:** 10.3390/antiox7040046

**Published:** 2018-03-24

**Authors:** Anita Umerska, Caroline Gaucher, Felipe Oyarzun-Ampuero, Isabelle Fries-Raeth, Florence Colin, María Gabriela Villamizar-Sarmiento, Philippe Maincent, Anne Sapin-Minet

**Affiliations:** 1 Université de Lorraine, CITHEFOR, F-54000 Nancy, France; anita.umerska@univ-lorraine.fr (A.U.); Isabelle.Raeth@univ-lorraine.fr (I.F.-R.); florence.colin88@gmail.com (F.C.); philippe.maincent@univ-lorraine.fr (P.M.); anne.sapin@univ-lorraine.fr (A.S.-M.); 2 Departamento de Ciencias y Tecnología Farmacéuticas, Facultad de Ciencias Químicas y Farmacéuticas, Universidad de Chile, 758-0150 Santiago, Chile; foyarzuna@ciq.uchile.cl (F.O.-A.); magabrielavillamizarsarmiento@gmail.com (M.G.V.-S.)

**Keywords:** curcumin, nanoparticles, PLGA, PCL, Eudragit, cytocompatibility, intestinal cells, single emulsion-solvent evaporation method

## Abstract

Despite the promising biological and antioxidant properties of curcumin, its medical applications are limited due to poor solubility in water and low bioavailability. Polymeric nanoparticles (NPs) adapted to oral delivery may overcome these drawbacks. Properties such as particle size, zeta potential, morphology and encapsulation efficiency were assessed. Then, the possibility of storing these NPs in a solid-state form obtained by freeze-drying, in vitro curcumin dissolution and cytocompatibility towards intestinal cells were evaluated. Curcumin-loaded Eudragit^®^ RLPO (ERL) NPs showed smaller particle diameters (245 ± 2 nm) and better redispersibility after freeze-drying than either poly(lactic-*co*-glycolic acid) (PLGA) or polycaprolactone (PCL) NPs. The former NPs showed lower curcumin encapsulation efficiency (62%) than either PLGA or PCL NPs (90% and 99%, respectively). Nevertheless, ERL NPs showed rapid curcumin release with 91 ± 5% released over 1 h. The three curcumin-loaded NPs proposed in this work were also compatible with intestinal cells. Overall, ERL NPs are the most promising vehicles for increasing the oral bioavailability of curcumin.

## 1. Introduction

Curcumin, or diferuloylmethane [1,7-bis(4-hydroxy-3-methoxyphenyl)-1,6-heptadiene-3,5-dione; molecular weight 368.37 g/mol] is a natural polyphenol isolated from the rhizome of turmeric (*Curcuma longa*), a member of the ginger family (*Zingiberaceae*). This popular spice is used for the treatment of inflammation, as a tonic and a blood purifier in traditional Indian (Ayurveda) and Chinese medicine [1,2,3]. Curcumin displayed antioxidant properties in vitro through free radical scavenging by the phenolic and methoxy groups on benzene rings and β-diketone [1,2,4]. This antioxidant property of curcumin explains many of its wide ranging pharmacological activities [1] in relation to pathologies associated with oxidative stress. Indeed, it has been reported that curcumin exhibits antidiabetic [1], anti-inflammatory [1,2,5], anti-hepatotoxic [6], anti-proliferative [1,7], anti-carcinogenic [1,7] and neuroprotective properties [2]. For instance, it has been shown that curcumin inhibits the expression of TNF-α-induced pro-inflammatory cytokines, such as IL-1β, IL-6 in TNF-α-treated HaCaT cells (immortalized human keratinocytes) and inhibits the expression of TNF-α-induced cyclin E [5]. The anti-inflammatory activity of curcumin can be linked to its ability to scavenge superoxide radicals, hydrogen peroxide, and nitric oxide produced by activated macrophages [4]. Curcumin is considered a potential agent for the treatment of inflammatory bowel disease (IBD) based on its anti-inflammatory and antioxidant activities. For instance, curcumin inhibits the activation of transcription factor NF-κB, cyclooxygenase 2 (COX-2) receptors and the p38 MAPK signaling pathway [8]. Curcumin has also been shown to exert an anticancer effect in human breast cancer cells (MCF-7) by inducing apoptosis and increasing caspase-3/9 activities in a time- and concentration-dependent manner via significant effects that were observed after 48-h exposure to 2 µM (0.74 µg/mL) of curcumin [7]. The key mechanism of the anticancer effect of curcumin was downregulation of miR-21 expression in MCF-7 cells by upregulating the PTEN/Akt signaling pathway [7].

The drawbacks of curcumin, such as low bioavailability, poor tissue distribution, extensive metabolism, chemical instability and potential for toxicity [9], have led to its classification as both a PAINS (pan-assay interference compound) and an IMPS (invalid metabolic panaceas) candidate. The main obstacles for potential medical applications of curcumin include insolubility in water (the reported solubility in deionized/distilled water at 25 °C is either 1.34 µg/mL [10] or 2.677 µg/mL [11]).

A possible solution to the above-mentioned obstacles is to use the nanocarriers. Nanocarriers may improve the bioavailability and therapeutic effectiveness of compounds poorly soluble in water, such as curcumin [12]. The concept of nanoarchitectonics is a powerful means to producing more advanced materials for biological applications [13]. A great variability of materials is available for the fabrication of nanoparticles, including inorganic materials such as metals, metal oxides, silica and organic materials such as low-molecular weight compounds, natural and synthetic polymers and biomacromolecules (proteins, nucleic acids) [13,14]. Polymeric nanoparticles have a range of advantages compared with other carriers. They are characterized by much better stability in the gastro-intestinal tract than, for example, liposomes and thus are capable of protecting the encapsulated drug. The incorporation of drugs into delivery systems in the form of nanoparticulate polymer matrices offers many benefits, e.g., controlled drug release and protection, prolonged blood circulation and many other adjustable characteristics [15]. Since the drug encapsulation within polymeric nanoparticles does not involve chemical modification of drug molecules but involves the drug-nanocarrier interactions by electrostatic forces and/or hydrophobic effects, it offers the advantage of minimization of additional biodegradability and toxicity studies.

Hydrophobic materials, such as poly-(lactic-*co*-glycolic acid) (PLGA) and polycaprolactone (PCL) commonly used for the fabrication of NPs seem to be appropriate carriers for curcumin. PLGA and PCL are both aliphatic polyesters synthesized by ring-opening polymerization. Both polymers, PLGA and PCL, have generated interest because of their excellent biocompatibility and biodegradability [16]. PLGA is degraded non-enzymatically in both earth environments and the human body, while PCL is enzymatically degraded in earth environments, but non-enzymatically in the body [17]. Both PLGA and PCL exhibit many properties beneficial for drug delivery systems. Therefore, they are promising candidates for the preparation of nanoscale drug delivery systems adapted to oral and parenteral administration [18]. Emulsion solvent evaporation and nanoprecipitation methods are commonly used to prepare PLGA and PCL nanoparticles [19]. For instance, curcumin-loaded PCL NPs have been shown to control the development of Ehrlich ascites carcinoma in in Swiss albino mice and increased their survival time [20]. In another study, Akl et al. [21] demonstrated that the curcumin uptake by colon adenocarcinoma cell line (HT29 cells) was enhanced after encapsulation within PLGA NPs compared with unformulated curcumin. These NPs displayed good colloidal stability in synthetic gastro-intestinal fluids and good long-term storage stability.

Eudragit^®^ RLPO (ERL) is a copolymer of ethyl acrylate, methyl methacrylate, approved by the US Food and Drug Administration and the European Medicines Agency. This non-biodegradable, water insoluble and positively-charged polymer is often used to prepare sustained-release formulations adapted to oral routes. Nanotechnology takes has taken advantage of ERL’s bioadhesive properties in the proposed drug delivery systems for oral [22,23], ocular [24] and intraarticular [25] administration. Owing to the above-mentioned properties, ERL is a promising material for preparing nanocarriers for oral delivery of curcumin. To the best of our knowledge, there have been no previous reports about the use of ERL nanoparticles for curcumin delivery. 

In this work, we attempted to formulate polymeric NPs, prepared via a standardized, single emulsion solvent evaporation method, adapted to the selected water insoluble polymers (PLGA, PCL and ERL). Physicochemical characterization of the nanocarriers was conducted to compare the properties of curcumin-loaded NPs: hydrodynamic diameter, size distribution, zeta potential, encapsulation efficiency and drug loading were investigated. In vitro kinetics of curcumin release, NPs recovery and redispersibility after freeze-drying (attractive approach for achieving long term stability) were also addressed and linked to in vitro cytocompatibility with intestinal cells.

## 2. Materials and Methods

### 2.1. Materials

Curcumin (78% purity by HPLC) was provided by Sigma-Aldrich (Saint Quentin-Fallavier, France). Poly(d,l-lactide-*co*-glycolide) (PLGA; Resomer^®^ RG 502 H) was obtained from Boehringer-Ingelheim (Ingelheim am Rhein, Germany). Polycaprolactone (PCL was purchased from Sigma-Aldrich (Saint Quentin-Fallavier, France). Eudragit^®^ RLPO was a gift from Evonik-IMCD (Darmstadt, Germany). Polymer specifications according to manufacturers’ data are presented in Table 1 and FTIR spectra of PCL, PLGA and ERL are shown in the Supplementary Materials (Figure S1). Poly(vinyl alcohol) (PVA, average molecular weight 30,000–70,000; 87–90% hydrolysed), MTT and cell culture reagents were provided by Sigma-Aldrich (Saint Quentin-Fallavier, France). All other chemicals and solvents were of analytical grade.

### 2.2. Curcumin Quantification

A 1.6 mg/mL curcumin stock solution was prepared in acetone and diluted 10-fold with either 0.5% aqueous solution of PVA (encapsulation studies) or 0.1% (*w*/*v*) Tween 80 in PBS (pH 7.4) (for solubility and release studies). Standard solutions of curcumin with concentrations ranging from 0.03 to 8 µg/mL were prepared by dilution with 10% acetone in either 0.5% PVA solution in water or 0.1% (*w*/*v*) Tween 80 in phosphate-buffered saline (PBS) (pH 7.4). Curcumin absorbance was measured at 425 nm or 430 nm in plastic cuvettes (Hellma), with 10% acetone used as a reference (UV-1601PC UV-visible spectrophotometer, Shimadzu, Kyoto, Japan). The wavelengths for absorbance measurements were selected based on the spectra.

### 2.3. Preparation of Nanoparticles

Blank and curcumin-loaded polymeric NPs were prepared using the emulsion-solvent evaporation method. Each polymer (250 mg) and optionally 25 mg of curcumin were dissolved in 5 mL of dichloromethane. The solution was then placed in a glass syringe and added dropwise to 10 mL of 0.5% (*m*/*v*) PVA solution under stirring (800 rpm). Stirring was continued for 10 s. Samples were then immersed in an ice bath and ultrasonicated for 3 min (13 W, 40% amplitude) using an ultrasonic probe (CV18) of 3 mm-diameter (ultrasonic processor, 130 W; 7501D, Fisher Scientific, Illkirch, France). The dispersion was then added to 40 mL of 0.5% (*m*/*v*) PVA solution and stirred at 800 rpm for 1 min. Dichloromethane was evaporated at 35 °C and NP dispersion was concentrated to a final volume of 10–14 mL (Büchi heating bath B-490, Büchi, Flawil, Switzerland) under reduced pressure (approximately 50 mBar) using a vacuum rotary evaporator (Büchi Rotavapor R-200, Büchi, Switzerland) equipped with condensor (Minichiller, Huber, Offenburg Germany) and pump Vacuum Controler CVC 2 (Vacuubrand, Wertheim, Germany).

NP dispersion was centrifuged at 42,000 *g* (Biofuge stratos, Hanau, Germany) for 20 min at 20 °C. Supernatants were recovered to perform the quantification of non-encapsulated curcumin (indirect method) by measuring the absorbance, as described in Section 2.2. The particles were re-suspended in water and frozen at −80 °C. Frozen samples were placed in a FreeZone 6 LABCONCO freeze-dryer (Labconco, Kansas City, Missouri, USA). A vacuum was obtained using a Vacuubrand RZ9 vacuum pump (Fisher Scientific, Illkirch, France). The freeze-dryer was operated in Auto Mode (collector temperature was −40 °C and pressure was equal or lower than 0.133 mBar). At the end of the freeze-drying cycle (24 h), the vials were removed from the machine and capped.

NP recovery was calculated using the following equation:

Y = (A/B) × 100%,(1)

where A is the mass of the NPs after freeze-drying, and B is the mass of polymer and optionally, the mass of curcumin used for NP preparation.

The encapsulation efficiency (EE) and drug loading (DL) were calculated using the following equations:

EE = ((C − D)/C) × 100%(2)

where C is the total amount (mass) of curcumin and D is the mass of non-encapsulated curcumin

DL = ((C − D)/E) × 100%(3)

where E is the total mass of all NP components.

Samples were reconstituted by dispersing an accurately weighed amount in water or cell culture medium.

### 2.4. Characterization of Nanoparticles

The intensity-averaged particle diameters and polydispersity indices of the NPs were determined by dynamic light scattering (DLS) with 173° backscatter detection. The electrophoretic mobility values, measured by laser Doppler velocimetry, were converted to zeta potential by the Smoluchowski equation. Both DLS and LDV measurements were performed on a Zetasizer nano series Nano-ZS fitted with a 633 nm laser (Malvern Instruments, Malvern, UK). The measurements were performed at an NP concentration of approximately 0.3 mg/mL, obtained by 30-fold dilution of 10 mg/mL dispersion with MilliQ water. Each analysis was carried out at 25 °C in triplicate.

Particle morphology was evaluated by scanning electron microscopy (SEM). A strip of double-sided carbon tape was placed on a SEM stub. A small quantity of freeze-dried NPs was gently spread across the surface of the tape, and compressed air was used to remove loose particles. Samples were sputter-coated with carbon for 20 s and viewed under a Hitachi S-4800 scanning electron microscope (Hitachi High-Technologies Corporation, Hitachi, Japan).

### 2.5. In Vitro Release of Curcumin from Nanoparticles

To ensure the sink conditions during the release studies, the solubility of curcumin in the release medium (0.1% *w*/*v* Tween 80 in PBS) was determined. An accurately weighed excess of curcumin (20 mg) was suspended in 20 mL of 0.1% (*w*/*v*) Tween 80 in PBS and incubated at 37 °C in a water bath at 200 rpm (Bioblock Scientific polystat circulation thermostat, Fisher Scientific, Illkirch, France) and Thermo Scientific Variomag^®^ Telesystem, Fisher Scientific, Illkirch, France). After 24 h and 48 h, 1 mL aliquots were withdrawn, transferred into microtubes and centrifuged at 42,000 *g* for 20 min at 20 °C. The quantity of dissolved curcumin was determined by absorbance measurement at 425 nm, as described in Section 2.2.

To evaluate the in vitro release kinetics, accurately weighed curcumin raw material, lyophilized blank (control) and curcumin-loaded NPs were suspended in 0.1% (*w*/*v*) Tween 80 in PBS (pH 7.4) to obtain a concentration equivalent to 10 µg/mL of curcumin. Aliquots of 1.2 mL were transferred into microtubes. Samples were incubated at 37 °C at 100 rpm in a shaking water bath (Memmert, Schwabach, Germany). After 5 min, 30 min, 1 h, 2 h, 4 h and 24 h, samples were centrifuged at 42,000 *g* for 20 min at 20 °C and 1 mL of supernatant containing the released curcumin was removed for curcumin quantification. Acetone (10% *v*/*v*) was added to the removed supernatant, and curcumin absorbance was measured at 425 nm, as described in Section 2.5. To compensate for the removed volume, 1 mL of release medium (Tween 80 0.1% *w*/*v* in PBS) was added to each microtube and NPs were re-dispersed using a micropipette. Samples were incubated until the next sampling time.

The data from the release studies were fitted to the first order equation:

(4) $$W=W_{\infty }(1-e^{-\mathrm{kt}})$$

where W is the amount of curcumin released at time t (based on cumulative release), W_∞_ is the amount of curcumin released at infinity and k is the release rate constant [26]. It was assumed that at the beginning of the release studies (t = 0 min), no curcumin was released (W = 0 µg/mg).

### 2.6. Intestinal Cells Cytocompatibility

Intestinal Caco-2 cells (ATCC^®^ HTB-37™, LGC Standards S.a.r.l. , Molsheim, France) were grown in complete medium, consisting of Eagle’s Minimum Essential Medium (EMEM) supplemented with 20 % (*v*/*v*) fetal bovine serum (FBS), 4 mM of glutamine, 100 U/mL of penicillin, 100 U/mL of streptomycin and 1 % (*v*/*v*) of non-essential amino acids. Cells were cultivated at 37 °C under 5 % CO_2_ (*v*/*v*) in a humidified incubator. Caco-2 cells with passage numbers ranging from 42 to 60 were seeded in 96-well plates at 20,000 cells/well, 24 h before experiment. NP cytocompatibility, expressed as metabolic activity, was evaluated by the 3(4,5-dimethylthiazol-2-yl)-2,5-diphenyltetrazolium bromide (MTT) assay. Caco-2 cells were exposed to either free curcumin (from 0.05 µg/mL to 50 µg/mL), or NPs loaded with curcumin (from 0.05 µg/mL to 50 µg/mL) that were dispersed in complete medium containing 0.1% (*v*/*v*) DMSO. The MTT solution (5 mg/mL) was then added into each well and incubated for 3 h at 37 °C. Formazan crystals were dissolved in DMSO under stirring and the absorbance was read at 570 nm with a reference at 630 nm (EL 800 microplate reader, Bio-TEK Instrument, Inc.^®^, Colmar, France). The cell viability was expressed as the ratio of the absorbance of treated cells to that of the negative control (complete medium added with 0.1% DMSO), which was assumed to have 100% cell viability.

### 2.7. Statistical Analysis

Results are showed as mean ± standard deviation, based on 3 experiments. The MTT results are expressed as mean ± standard error of mean, based on 3 experiments. The statistical significance of the differences between samples was determined using GraphPad Prism 5 software with either one-way analysis of variance (ANOVA) (for encapsulation efficiency, drug loading, NP recovery, and kinetics of curcumin release data) or two-way ANOVA (for particle size, polydispersity index, zeta potential and cytocompatibility data) with Bonferroni post-test. Differences were considered significant at *p* < 0.05.

## 3. Results and Discussion

### 3.1. Emulsification Solvent Evaporation Method

There are many techniques that can be used to produce NPs, which allows for broad differentiation of their internal and external structure, composition and biological properties [27]. In general, polymeric nanoparticles may be prepared either by polymerization of dispersed monomers or by dispersion of pre-formed polymers [16]. The use of particles obtained by polymerization may be limited because of the presence of residual monomers, oligomers, catalysts and possible cross-reaction with drugs [28]. Due to the chemical reactivity/instability of curcumin, a method of NP preparation that involves dispersion of preformed polymers is more appropriate than polymerization. There are a few preparation methods of NPs from polymers, the choice of which is highly dependent on the hydrophobic/hydrophilic characteristics and solubility of polymers and the active ingredients to be incorporated [19].

**Figure 1:**
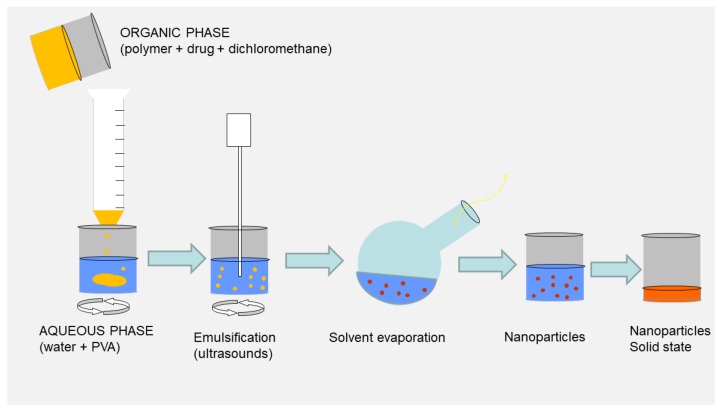
Schematic diagram of the emulsion solvent evaporation method.

Here, we describe the emulsion solvent evaporation (ESE) method, involving the use of dichloromethane as a solvent and PVA as a surfactant. Ultrasonication was used as the emulsification method. A schematic diagram of the ESE process is shown in Figure 1. This protocol has been optimized in our laboratory for PLGA NPs based on Akl et al. [21]. All process parameters, such as quantities of excipients, stirring speed, sonication time and intensity, were kept constant for all types of polymeric NPs in order to evaluate the influence of polymer type and/or drug loading on the properties of the NPs.

### 3.2. Formation and Characteristics of Blank and Drug-Loaded Polymeric Nanoparticles

The first characteristic that should be performed for nanoparticle carriers is the evaluation of macroscopic aspects/homogeneity by visual observation of the sample. All samples appeared as turbid, but macroscopically homogenous. Blank NP formulations were milky-white or opaque. The Tyndall effect was easily observed in ERL NPs, and also in PLGA NPs. It was more difficult, but possible to observe the Tyndall effect in PCL NPs. Under the Tyndall effect, the long wavelengths are transmitted to a higher extent than the short wavelengths, and the latter are scattered more strongly. The Tyndall effect is observed in nano-suspensions when the diameters of dispersed particles are in the range from 40 nm to 900 nm. Interestingly, all curcumin-loaded formulations showed different colors. Dispersions of curcumin-loaded ERL, PLGA and PCL NPs were red, yellow-orange and yellow, respectively. Curcumin demonstrates keto-enol tautomerism. The bis-α, β-unsaturated β-diketone exists in equilibrium with its enol tautomer [29]. These forms have different colors. The bis-keto form predominates in acidic and neutral aqueous solutions and in the cell membrane, whereas the enol form is in the majority of aqueous solutions at pH above 8 and in the solid state. The first pKa (7.5 to 8.5) changes curcumin color from yellow to red. In the pH range between 3 and 7, curcumin acts as a potent donor of hydrogen atoms, while, at pH 8, this compound acts mainly as an electron donor, similar to many phenolic antioxidants. The α,β-unsaturated carbonyl is a good Michael acceptor and undergoes nucleophilic addition [29,30,31,32]. Diverse colors of different types of polymeric NPs suggest that the quantities of the keto and enol tautomers are different in all these formulations. Because the color of curcumin depends on pH [32], the pH of NP dispersions were measured. The pH of PLGA NPs was 3.7–3.85. The anions of acidic groups of PLGA may be proton acceptors, and this may facilitate the ionization of a small fraction of curcumin molecules, which can serve, in this case, as proton donors and may explain the yellow-orange color, which suggests the presence of both, keto and enol forms. PCL NPs had pH 5.25–5.55. PCL is a non-charged hydrophobic polymer, and, in this hydrophobic environment, the keto form seems to predominate. The red color observed in curcumin-loaded ERL NPs was similar to that observed at basic pH (9.5–10) by Pourreza and Golmohammadi [32], but the measured pH, rather than being basic as would be expected, was in the range 5–5.2. It is at basic pH that curcumin exists in the anionic form [30]. However, due to the fact that ERL is a cationic polymer, it may change the conformation of curcumin and promote ionization of curcumin to produce electrostatic interactions between the anionic form of curcumin and cations of quaternary ammonium groups of ERL. When mixed with acetone, the supernatant from centrifugation of curcumin-loaded ERL NPs containing the non-encapsulated curcumin fraction became yellow, whereas the NP fraction, after mixing with acetone, aggregated and the color became orange-brown. Because of the variable color of different samples, it was necessary to dilute them as much as 1000 times during quantification by UV to provide the same environment so that all the samples would produce yellow color similar to that of the standard.

Another very important parameter which must be taken into account when formulating nanoparticles is the possibility of obtaining a solid state form. As colloidal systems, NPs are thermodynamically unstable and may be susceptible to aggregation after extended periods of storage as a suspension. Long term stability is an important challenge in the development of NPs. Moreover, solid state form may improve the stability of substances that are prone to degradation and unstable in an aqueous environment. Freeze-drying is an attractive approach for achieving long term stability, as suspensions can be converted into solid state materials with greater physical stability than liquids. Lyophilized formulations also provide easy handling including during shipping and storage [33]. However, it has been shown that stress generated during freeze-drying may adversely impact the properties of NPs [34]. For this reason, the redispersibility of polymeric NPs after freeze-drying was evaluated. Lyophilized particles showed a cotton-like texture. Blank NPs had a white or grey color; curcumin-loaded ERL, PLGA and PCL NPs powder appeared red, orange-yellow and yellow-brown, respectively. After reconstitution of each formulation in water the color was the same as that before freeze-drying.

**Figure 2:**
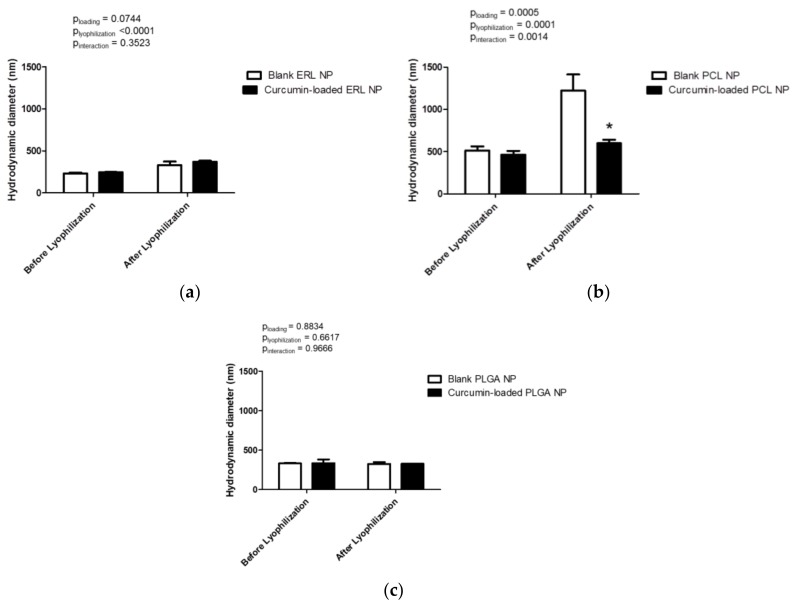
Hydrodynamic diameters of blank and curcumin-loaded polymeric nanoparticles before and after freeze-drying: (**a**) Eudragit^®^ RLPO (ERL) nanoparticles (NPs) (**b**) polycaprolactone (PCL) NPs and (**c**) poly-(lactic-*co*-glycolic acid) (PLGA) NPs. Results are expressed as mean values (*n* = 3) ± standard deviation and compared using two-way ANOVA with Bonferroni post-test; * *p* < 0.05 versus blank NPs after lyophilization.

The particle size affects the biopharmaceutical properties of the nanocarriers and drug release [35]. DLS measurements confirmed that polymeric nanoparticles with sizes in the range of hundreds of nanometers were successfully obtained using the single emulsion-solvent evaporation method (Figure 2). ERL NPs showed the smallest particle size (231 ± 8 nm) of all tested polymers (Figure 2a). Curcumin loading did not exert a significant effect on particle size (*p* = 0.0744). Although ERL NPs were successfully re-dispersed after freeze-drying, a significant increase in particle size, by 100–120 nm, for both blank and curcumin-loaded NPs was observed (*p* < 0.0001). The largest particle size was obtained for PCL NPs (512 ± 50 nm) (Figure 2b). Both lyophilization and curcumin loading affected the particle size of PCL NPs (*p* = 0.0001 and *p* = 0.0005, respectively). After lyophilization of blank PCL NPs, the particle diameter was doubled and the presence of a small quantity of aggregates was observed. Interestingly, incorporation of curcumin improved the redispersibility of drug-loaded PCL NPs compared with blank PCL NPs. The re-dispersed curcumin-loaded particles showed an average size of 641 nm and were macroscopically homogenous. PLGA NPs were characterized by a diameter of 332 ± 7 nm (Figure 2c). Although the size of reconstituted blank and curcumin-loaded PLGA NPs was not markedly different from that before freeze-drying (p_lyophilization_ = 0.6617, p_loading_ = 0.8834 and p_interaction_ = 0.9666), the presence of a small quantity of aggregates was observed. These aggregates did not interfere with DLS measurements due to rapid sedimentation. The DLS measurements are in agreement with the visual aspect of the samples and observation of the Tyndall effect.

**Figure 3:**
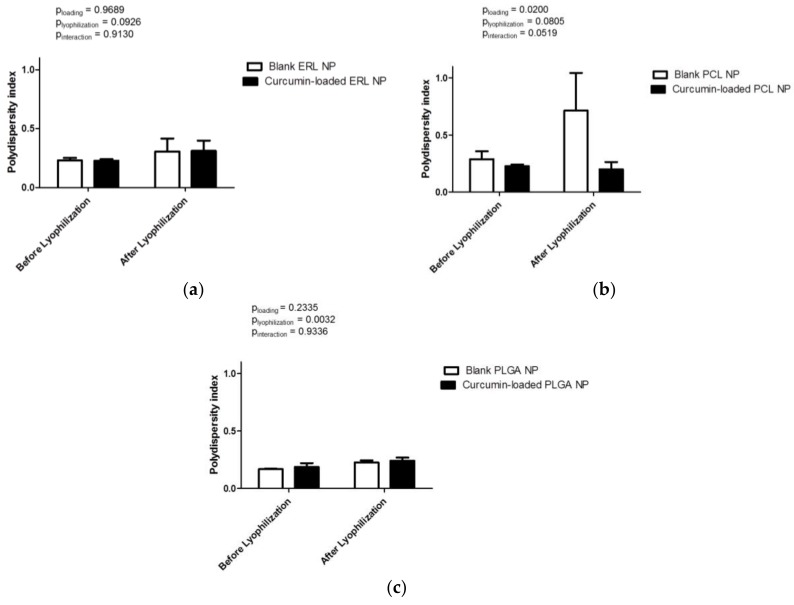
Polydispersity index of blank and curcumin-loaded polymeric nanoparticles before and after freeze-drying: (**a**) ERL NPs, (**b**) PCL NPs and (**c**) PLGA NPs. Results are expressed as mean values (*n* = 3) ± standard deviation and compared using two-way ANOVA.

The polydispersity index is the ratio of size deviation (or width of size distribution) to mean particle diameter [35]. High polydispersity index values indicate large variations in particle size, whereas polydispersity index values smaller than 0.3 suggest that particles are monodisperse. All formulations were characterized by homogenous size distribution with polydispersity index values in the range between 0.169 ± 0.004 (blank PLGA NPs) and 0.289 ± 0.069 (blank PCL NPs) (Figure 3). Neither curcumin loading (*p* = 0.9689), nor freeze-drying (*p* = 0.0926), affected the size distribution of ERL NPs (Figure 3a). Curcumin loading significantly decreased the polydispersity index of PCL NPs (*p* = 0.0200) (Figure 3b). Lyophilization did not have a significant effect on the size distribution of PCL NPs (*p* = 0.0805). However, the polydispersity index of blank PCL NPs after freeze-drying should be interpreted with caution because high values of this parameter and the presence of aggregates indicate that the sample is not suitable for DLS analysis. The improvement of the redispersibility of PCL NPs by curcumin loading is reflected not only by a lower particle diameter, but also by a more homogenous size distribution. Curcumin loading did not affect the size distribution of PLGA NPs (*p* = 0.2335) (Figure 3c). Freeze-drying slightly increased the polydispersity index of PLGA NPs (*p* = 0.0032). 

**Figure 4:**
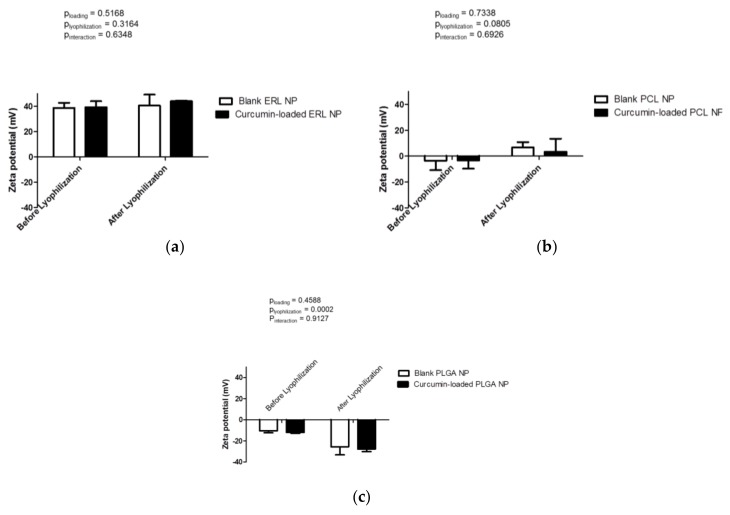
Zeta potential of blank and curcumin-loaded polymeric nanoparticles before and after freeze-drying: (**a**) ERL NPs, (**b**) PCL NPs and (**c**) PLGA NPs. Results are expressed as mean values (*n* = 3) ± standard deviation and compared using two-way ANOVA.

The zeta potential data of blank NPs reflects the charges of the raw polymers. Indeed, the polycationic Eudragit RL, bearing positive charges, conferred by the quaternary ammonium groups (8.8–12%), presented the highest zeta potential (+38.7 ± 4.0 mV) (Figure 4a). The zeta potential of ERL NPs was not influenced either by curcumin loading (*p* = 0.5168) or by lyophilization (*p* = 0.3164). As reported many times, PCL and PLGA, being mostly uncharged polymers, displayed negative zeta potential values, close to neutrality. PCL NPs can be considered to be neutral to the zeta potential of −3.6 ± 7.3 mV (Figure 4b). This is not surprising because the PCL molecules do not contain any ionizable groups in their structure. The zeta potential was not modified either by lyophilization (*p* = 0.0805) or by curcumin loading (*p* = 0.7338). However, due to the limitations of laser Doppler velocimetry, the neutral zeta potentials are only estimated values and should be interpreted with caution. PLGA NPs exhibited a slightly negative zeta potential of −10.5 ± 1.8 mV (Figure 4c), which may be due to the ionization of lactic and/or glycolic acid moieties in PLGA molecules. Curcumin loading did not influence the zeta potential of polymeric NPs (*p* = 0.4588). Interestingly, the zeta potential in modulus of re-dispersed PLGA NPs increased significantly after freeze-drying (*p* = 0.0002). This may be attributed to the rearrangement in surface coverage by PVA molecules and possibly the removal of some PVA molecules from the NP surface and larger exposure of anionic groups of PLGA to the external environment.

It has been reported that in order to effectively stabilize the NPs during freeze-drying and to ensure their adequate reconstitution, suitable excipients are required [34]. It is possible that PVA plays the role of a stabilizing agent and cryoprotectant during the lyophilization of NPs. PVA is an emulsion stabilizer and stabilizes the polymeric NPs not only during the emulsification step and in solution, but also in the solid state. PVA is known to bind to the surface of PLGA NPs in an irreversible manner. The PVA binding affects the hydrophilicity/hydrophobicity of a particle’s surface and its digestibility [36]. Boury et al. [37] have shown that PLGA microparticles, surface coated with PVA, displayed higher water wettability compared with a reference hydrophobic PLGA film surface. Thus, the PVA adsorption may enable wetting and facilitate the redispersion of polymeric NPs after lyophilization. The addition of other cryoprotectants should be considered for more effective freeze-drying, particularly in the cases of PLGA and PCL NPs.

**Figure 5:**
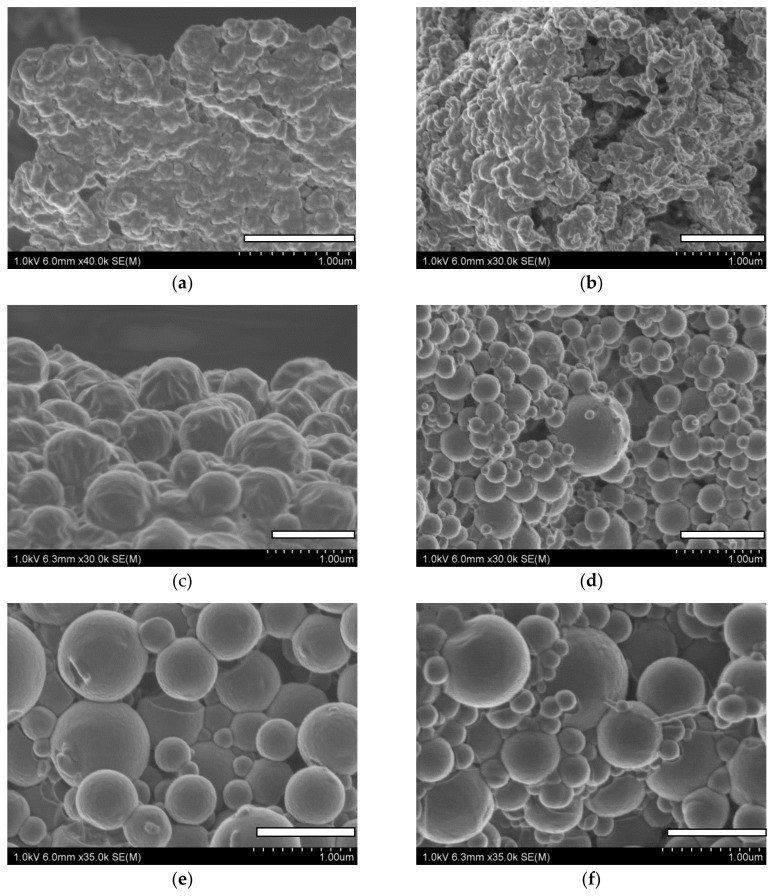
SEM of (**a**) blank ERL NPs, (**b**) curcumin-loaded ERL NPs, (**c**) blank PCL NPs, (**d**) curcumin-loaded PCL NPs, (**e**) blank PLGA NPs and (**f**) curcumin-loaded PLGA NPs. Scale bars: 1 µm.

NP morphology was examined by SEM. Both blank and curcumin-loaded ERL NPs appeared as fused particle assemblies (Figure 5a,b). Because samples were successfully re-dispersed after freeze-drying, as indicated by DLS measurements and visual observation; the presence of poorly formed particles in SEM images can possibly be explained by particle fusion during sputter coating. Moreover, local heating from the SEM beam was found to destroy the particles and alter their morphology to a higher extent than in the case of either PCL or PLGA NPs. PLGA and PCL NPs had spherical shapes (Figure 5c–f); however, in some cases, deformations were observed due to the presence of neighboring particles. The particles generally appeared as either individual particles or assemblies of non-fused particles; some of them were connected by “bridges”, with a broad range of sizes distributed throughout the sample. The particles displayed a rough surface. No shape modification was observed in the presence of curcumin, independent of the polymer used (Figure 5b,d,f).

### 3.3. Nanoparticle Recovery, Curcumin Loading and Encapsulation Efficiency

The formulation of NPs involved a purification step and other operations that require material transfer, which can lead to possible loss of NPs. For this reason, it is important to determine NP recovery, also referred to as NP yield. To calculate the NP recovery, only the mass of polymer used for the fabrication of NPs and, in the case of curcumin-loaded particles, the mass of curcumin, was taken into account. NP recovery is shown in Table 2. Curcumin loading did not affect the NP yield. Approximately 64–67% of PCL and PLGA NPs were recovered. The production yield of ERL NPs was significantly smaller compared with both PLGA and PCL. The smaller yield observed for ERL may be explained by the presence of an important fraction of NPs with a small size that did not undergo sedimentation during the centrifugation process. Indeed, the DLS measurements confirmed that ERL NPs were characterized by the smallest size. Moreover, ERL contains hydrophilic ammonium groups that are present as salts and make this polymer more permeable to water [38]. Thus, the possible penetration of water into the particle structure can affect particle density and decrease the difference in density between the particles and the external aqueous phase, thereby reducing the sedimentation rate.

NP recovery was determined by dividing the mass of NPs by the mass of polymer carrier and curcumin, whereas drug loading was defined as a ratio of curcumin mass divided by the mass of the polymer carrier and curcumin (Section 2.3). It is likely that the NPs also contain PVA that was not taken into account in the determination of either DL or NP recovery. It has been demonstrated that PLGA particles with a size below 1 µm have a constant PVA surface density of about 1.8 mg/m^2^ that is independent of the PVA concentration in the continuous phase of the manufacturing process. The PVA contents were approximately 24 mg/g and 28.2–32.5 mg/g for 0.53 µm and 0.35–0.37 µm particles, respectively [36].

When developing an effective nanoparticulate delivery system, drug loading is a key parameter, as low loading often limits the use of such systems, because a substantial amount of the formulation must be administered to achieve the therapeutic effect. For this reason, high drug encapsulation efficiency is important for developing successful formulations. The type of polymer had an important, statistically significant effect on curcumin encapsulation. PCL NPs were characterized by the highest EE from all tested polymers, with 99 ± 0.2% of curcumin incorporated within the NPs. The EE in PLGA NPs (90 ± 1.5%), although significantly smaller than that of PCL NPs, can also be considered very high. Interestingly, the EE was markedly reduced in the case of ERL NPs to 61.7 ± 0.7%. The theoretical loading for all drugs should be 9.1%. The loading obtained for PCL and PLGA NPs are close to this value; however, the loading in ERL NPs was considerably smaller (5.61 ± 0.43%). This is in good agreement with the encapsulation efficiency. There are a few possible explanations for the lowest EE and loading observed for ERL NPs. The affinity of curcumin to ERL may be lower than the affinity to PLGA or PCL. The considerably low yield of ERL NPs may contribute to a low EE because a significant fraction of curcumin could have been lost with the ERL polymer. Another possible explanation for the lower encapsulation of curcumin in ERL NPs could be the fact that some of curcumin molecules underwent the aza-Michael addition to quaternary ammonium groups of ERL. The aza-Michael addition (conjugate reaction of various amines with α,β-unsaturated carbonyl compounds that provides β-amino carbonyl ingredients) does not require a basic catalyst and can occur in water, in contrast with the ‘classic’ Michael reaction. Nevertheless, even ERL NPs were capable of encapsulating quantities of curcumin that were high enough to produce curcumin concentration of at least 500 µg/mL after reconstitution. This concentration is more than 500 times higher than that reported to exert anticancer effects (0.74 µg/mL) against MCF-7 cells [7]. 

### 3.4. In Vitro Release Studies

**Figure 6:**
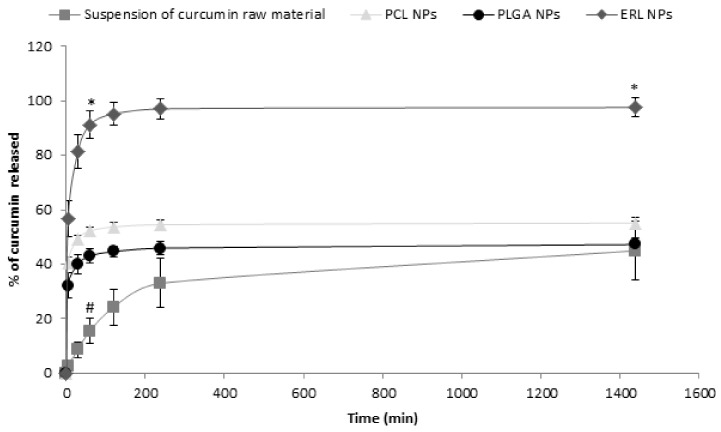
Cumulative release profiles of curcumin from curcumin-loaded NPs and suspension of curcumin raw material into PBS. The experiments were carried out at 37 °C. Results are expressed as mean values (*n* = 3) ± standard deviation and compared using one-way ANOVA with Bonferroni post-test; * *p* < 0.05 versus PCL NPs, PLGA NPs and suspension of curcumin raw material; ^#^ *p* < 0.05 versus PCL NPs and PLGA NPs.

The in vitro release studies of curcumin from polymeric NPs were performed to determine the influence of the polymer on the release kinetics. Among the three tested polymers, only ERL NPs released the total amount of encapsulated curcumin, whereas, in the case of either PCL or PLGA NPs, only 55 ± 2% and 47 ± 2% of curcumin, respectively, were released after 24 h (Figure 6). PLGA and PCL NPs showed the same kinetics of curcumin release, as there was no statistically significant difference either in the percentage of released curcumin at any time point or in the release rate constant (Table 3). A burst release was observed, with about 43 ± 3% and 52 ± 1% of curcumin released from PLGA and PCL NPs, respectively, within the first hour. After an initial burst release phase, only a very small amount of curcumin (less than 2% and 5% for PCL and PLGA NPs, respectively) was released between 1 h and 24 h. It is generally accepted that the initial phase of rapid drug release from NPs is the fraction weakly bound to or adsorbed on the NP surface. Then, the delayed release is probably due to drug diffusion from the matrix and/or matrix erosion [16]. The retention of curcumin in the PCL and PLGA NPs may be explained by the high affinity of this hydrophobic compound to those polymers. The retention of hydrophobic active pharmaceutical ingredients, such as promazine or chlorpromazine, in the nanocarriers has already been reported. The percentage of the compound that remained within the nanocarrier depended on its affinity to the nanocarrier and the entrapment efficiency [39]. It was impossible to extract the curcumin that remained in PCL NPs after release studies. A quantity of 77 ± 5% of curcumin was recovered from PLGA NPs (this includes the released curcumin and curcumin that was extracted from the NPs after release studies using acetone (dilution 1:1) and dichloromethane (dilution 1:10)).

ERL NPs showed a different release profile than the other polymers, because the percentage of released curcumin was markedly different from that released from either PCL or PLGA NPs at any time point (*p* < 0.0001). The release of curcumin from ERL NPs can be considered to be immediate, with 57 ± 7%, 81 ± 6% and 91 ± 5% of curcumin released after 5 min, 30 min and 60 min, respectively. The fact that the mass balance was obtained and the total amount of curcumin was recovered may indicate that the aza-Michael reaction either did not occur or was reversible. The higher percentage of released curcumin observed for ERL NPs may be explained by the fact that this polymer, although generally considered to be hydrophobic, contains the ammonium groups that are present as salts, making this polymer permeable to water [38]. This may facilitate the penetration of the release medium through the polymer matrix, thereby facilitating curcumin release. The release of a higher percentage of curcumin from ERL NPs can be linked with its lower EE compared with PLGA or PCL NPs. Moreover, the fact that ERL NPs released the total amount of encapsulated curcumin may compensate for the lower EE, as indicated by the amount of curcumin released at infinity.

Interestingly, the suspension of curcumin raw material displayed the lowest release rate, with the constant k significantly smaller than that observed for the NP formulations. Curcumin was released gradually and no burst release was observed. The percentage of curcumin released from the suspension of raw material was significantly lower than that released from NPs up to 2 h (*p* < 0.0001). The slow release from suspension may be attributed to the large particle size (particles were visible to naked eye) and rapid sedimentation of suspended curcumin, which settled on the bottom of Eppendorf tube and was more difficult to mix than the NPs. Moreover, the contact area of the curcumin suspension with the external aqueous phase was much smaller than that of NPs due to the smaller surface-to-volume ratio. As a hydrophobic material, curcumin probably shows lower wettability than the NPs covered by the surfactant (PVA). It has been reported that the dissolution rate of curcumin from dripping pills is higher than that of pure curcumin because the wettability and solubility of curcumin are improved by surfactants (Poloxamer 188 and Cremophor RH40) [40].

To ensure sink conditions during the release studies, the solubility of curcumin in 0.1% *w*/*v* Tween 80 in PBS was evaluated. The solubility of curcumin after 24 h at 37 °C was 28 ± 5 µg, and it did not change significantly after 48 h. The kinetics of curcumin release in our study were markedly different than those observed in PLGA NPs by Khalil et al. [41] , who observed prolonged release over a period of 9 days. The difference may be attributed to experimental conditions. Khalil et al. used PBS (0.01 M, pH 7.4) without the addition of surfactants, such as Tween 80, that could potentially increase the solubility of curcumin. Moreover, they used a relatively high concentration of curcumin (1.5 mg in 12 mL of PBS). This concentration is markedly higher than the solubility of curcumin, and, for this reason, the sink conditions were probably not achieved. In another study by Kasinathan et al. [20], the release of curcumin from PCL NPs has been described. After an initial burst release, the amount of released drug was low and steady. The initial burst release was attributed to the curcumin adsorbed on the NP surface. Although, in their study, the release was more prolonged than in our study, it is very unlikely than the sink conditions were achieved because NPs containing 2.5 mg of curcumin were transferred into 2 mL of PBS [20].

According to Biopharmaceutics Classification System (BCS), curcumin belongs to class II, with low solubility in water but high intestinal permeability [40]. Therefore, rapid release is a desired characteristic for oral administration of this compound, because enhancement of the dissolution rate could increase its bioavailability which is limited by poor solubility. 

### 3.5. Cytocompatibility Studies

**Figure 7:**
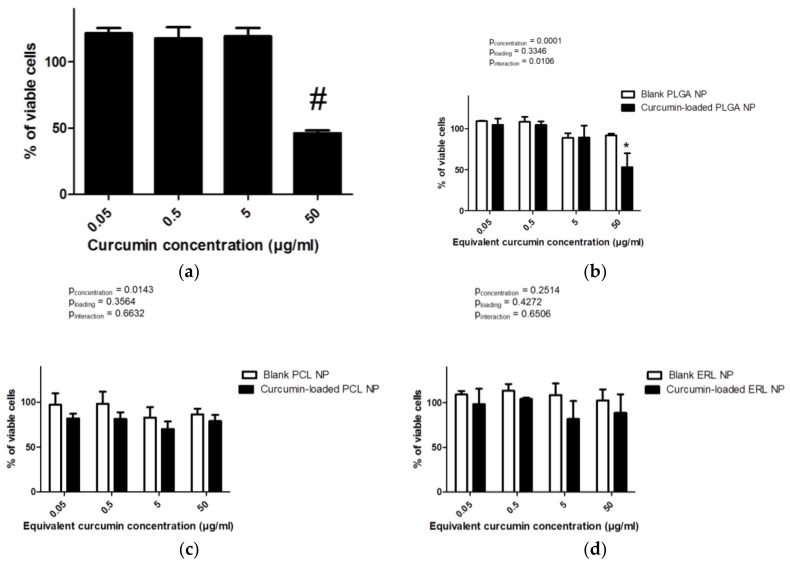
Cytotoxicity of curcumin solution (**a**), blank and curcumin-loaded PLGA NPs (**b**), PCL NPs (**c**) and ERL NPs (**d**) in Caco-2 cells after 24 h exposure. The quantities of particles that contained 50 µg of curcumin were 610 µg, 554 µg and 891 µg of PLGA, PCL and ERL, respectively. Results are expressed as mean values (*n* = 3) ± standard error of mean and compared using either one-way ANOVA (curcumin solution) two-way ANOVA (NP formulations) with Bonferroni post-test; ^#^ *p* < 0.05 versus curcumin at 0.05, 0.5 and 0.5 µg/mL, * *p* < 0.05 versus blank NPs.

The cytocompatibility of free curcumin, blank and curcumin-loaded polymeric NPs to intestinal Caco-2 cells was evaluated using the MTT assay. In living cells, mitochondrial dehydrogenase oxidizes the MTT to formazan product. Damaged or dead cells show reduced or no dehydrogenase activity [42,43]. It is generally accepted that if cell viability is higher than 80%, the compound can be considered as non-toxic to cells [42,43]. In our study, free curcumin, at a concentration equal to or lower than 5 µg/mL, did not affect the viability of Caco-2 cells (Figure 7a). However, at a concentration of 50 µg/mL (135.7 µM), free curcumin significantly impacted the metabolic activity of Caco-2 cells (46 ± 2% of viable cells) (*p* < 0.0001). It was impossible to test higher concentrations of curcumin, because curcumin absorbance interfered with the MTT test. These results confirmed those obtained by Wahlang et al. [42], showing the toxicity of curcumin at 265 µM (98 µg/mL) with only 50% Caco-2 cell viability after 24 h of exposure.

Blank NPs did not have any significant effect on the viability of Caco-2 cells with 92.0 ± 2.1%, 86.5 ± 6.3% and 102.5 ± 12.6% cell viability for PLGA, PCL and ERL NPs, respectively, at the highest equivalent curcumin concentration tested (Figure 7b–d). It is known that polymers that are insoluble in water generally do not interact physically or chemically with living cells unless the surface of the material has very sharp projections or a high density of cationic moiety. However, as a result of the degradation, biodegradable polymers always release low molecular weight compounds into the outer environment, and, in some cases, the physiology of the cell is disturbed by these foreign compounds [44]. The degradation products of PLGA or PCL are generally considered to be non-toxic. Among tested polymers, only ERL has a high density of cationic moiety. Although the zeta potential of ERL NPs in water was highly positive, it dropped drastically in complete medium with −5.81 ± 0.55 mV and −4.68 ± 0.35 mV for blank and curcumin-loaded ERL NPs, respectively. It has been shown that the charge of chitosan-based particles decreased or even inverted from highly positive to slightly negative in media with high ionic strength and after an increase in pH from slightly acidic to 7.4 [17,45]. Such a change in NP charge from positive to negative as a result of pH modification reduced the cytotoxicity of chitosan NPs towards Caco-2 cells [45]. Furthermore, a change in pH from 5 to 7.4 and an increase in the ionic strength of the medium might reduce the ionization of quaternary ammonium groups of ERL molecules. Moreover, positively charged particles can be surrounded by negatively charged components of the medium and this can also affect their zeta potential and interaction with cells. Furthermore, it has been shown that adsorption of albumin onto ERL NPs results in a considerable increase in particle size and polydispersity index [23].

Apart from PLGA NPs, no statistical differences were shown between blank or curcumin-loaded NPs showing that NPs are protective against toxicity from high concentrations of curcumin. The delayed release of curcumin from PCL NPs could explain the decrease in the cytotoxicity of this compound. In the case of long lasting releasing NPs, curcumin was presented gradually in terms of concentration and time allowing cells to adapt to stress conditions. However, the cytotoxicity and the interaction between cells and curcumin-loaded-NPs depend also on particle–cell interactions and particle uptake by cells.

## 4. Conclusions

It is important to define the criteria for the selection of nano-formulations with the most favorable properties. Many criteria should be taken into account, such as encapsulation efficiency, drug loading and the release of encapsulated molecules, physical properties (size, size distribution, zeta potential), the ability to obtain solid state form and redispersibility and cytocompatibility. There were no significant differences in cytotoxicity between the various NP formulations towards intestinal cells, even at concentrations markedly higher than those producing a desired effect on cells in vitro. Consequently, cytotoxicity is not a good criterion for formulation selection.

The advantages of PLGA and PCL NPs are high encapsulation efficiency and drug loading, but these formulations showed important disadvantages, such as problems with redispersibility after freeze-drying. Another important drawback of these formulations is that a significant amount of curcumin was not released.

The advantage of ERL is the rapid release of the total quantity of curcumin, which could possibly improve the bioavailability of this compound. ERL NPs are also promising carriers for the oral delivery of curcumin due to mucoadhesive properties of ERL. Nonetheless, the disadvantage of ERL NPs is low particle recovery and encapsulation efficiency. The ESE method is generally applied for polymers such as PLGA and PCL and some modifications may be needed to adjust it to ERL to improve the encapsulation efficiency and recovery of ERL NPs, possibly by removing purification steps and optimizing the volume of the aqueous phase. Moreover, it is possible to use the mixture of polymers ERL and PLGA and to investigate other types of Eudragit polymers. On the other hand, PLGA and PCL NPs could be considered as carriers for parenteral administration of curcumin. The NPs developed and characterized in the present study could potentially make a promising delivery system adapted to various administration routes.

## Figures and Tables

**Table 1:** Physicochemical characteristics of polymers used in the study (data obtained from manufacturers); N/A: not available, N/R: not relevant, DS: dry substance.

Polymer	ERL	PLGA	PCL
Formula	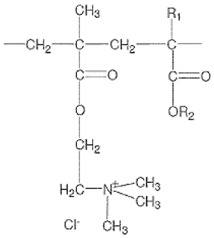	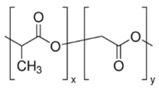	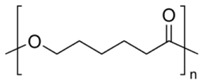
Average molecular weight	*M*_w_ = 150,000 g/mol	*M*_w_ = 40,000 g/mol	*M_n_* = 10,000 g/mol *M*_w_ = 14,000 g/mol
Viscosity	Max 15 mpa·s ^a^	0.22 dL/g ^b^	758 mPa·s ^c^
Acid value	N/A	9 mg KOH/g	0.2 mg KOH/g
Alkali value	23.9–32.3 mg KOH/g	N/A	12.6 mg KOH/g
Ammonio methacrylate units on DS	8.85–11.96%	N/R	N/R
% of d,l-lactide and glycolide units	N/R	50 mol % of d,l-lactide and 50 mol % glycolide ^d^	N/R

^a^ The viscosity of the test solution (12.5% *w*/*w* dry substance dissolved in a mixture of 60% *w*/*w* isopropyl alcohol and 40% *w*/*w* acetone) was determined by a Brookfield viscometer (UL adapter/30 rpm/20 °C); ^b^ Measured at 25 °C at 0.1% in CHCl_3_; ^c^ 50 wt %, xylene, at 25 °C; ^d^ by NMR spectroscopy.

**Table 2:** Nanoparticle recovery, encapsulation efficiency and drug loading of blank and curcumin-loaded polymeric nanoparticles. Results are expressed as mean values (*n* = 3) ± standard deviation and compared using one-way ANOVA with Bonferroni post-test. * *p* < 0.05 versus PLGA NPs, ^#^ *p* < 0.05 versus PCL NPs.

Formulation	NP Recovery	Encapsulation Efficiency	Drug Loading
Blank PLGA NPs	66 ± 9%	N/A	N/A
Blank PCL NPs	67 ± 5%	N/A	N/A
Blank ERL NPs	34 ± 4% *^,#^	N/A	N/A
Curcumin-loaded PLGA NPs	66 ± 16%	90.0 ± 1.5%	81.8 ± 1.4 mg/g
Curcumin-loaded PCL NPs	64 ± 2%	99.2 ± 0.2%	90.2 ± 0.2 mg/g
Curcumin-loaded ERL NPs	35 ± 6% *^,#^	61.7 ± 4.7% *^,#^	56.1 ± 4.3 mg/g *^,#^

**Table 3:** Model parameter estimates for curcumin release data fitted to the first-order model, where W_∞_ is the amount of curcumin released at infinity and k is the release rate constant. Data was compared using one-way ANOVA with Bonferroni post-test; * *p* < 0.05 versus curcumin raw material, ^#^ versus ERL NPs.

Sample	K (h^−1^)	W_∞_ (µg/mg)	Goodness of Fit (R^2^)
Suspension of curcumin raw material	0.0060 ± 0.0004	44.6 ± 10.8	0.8960
PCL NPs	0.288 ± 0.022 *^,#^	47.8 ± 1.5	0.9854
PLGA NPs	0.264 ± 0.052 *	36.1 ± 1.9 ^#^	0.9607
ERL NPs	0.185 ± 0.041 *	52.0 ± 2.3	0.9646

## Supplementary Materials

The following are available online at http://www.mdpi.com/2076-3921/7/4/46/s1, Figure S1: FTIR spectra of (**a**) PCL (**b**) PLGA and (**c**) ERL.
