# Supplementary material for: Polymeric Nanoparticles for Increasing Oral Bioavailability of Curcumin
Source: Antioxidants (Basel). 2018 Mar 24;7(4):46. doi: 10.3390/antiox7040046 (PMC5946112; doi:10.3390/antiox7040046)
Supplement: Supplementary file 1 [file antioxidants-07-00046-s001.pdf]

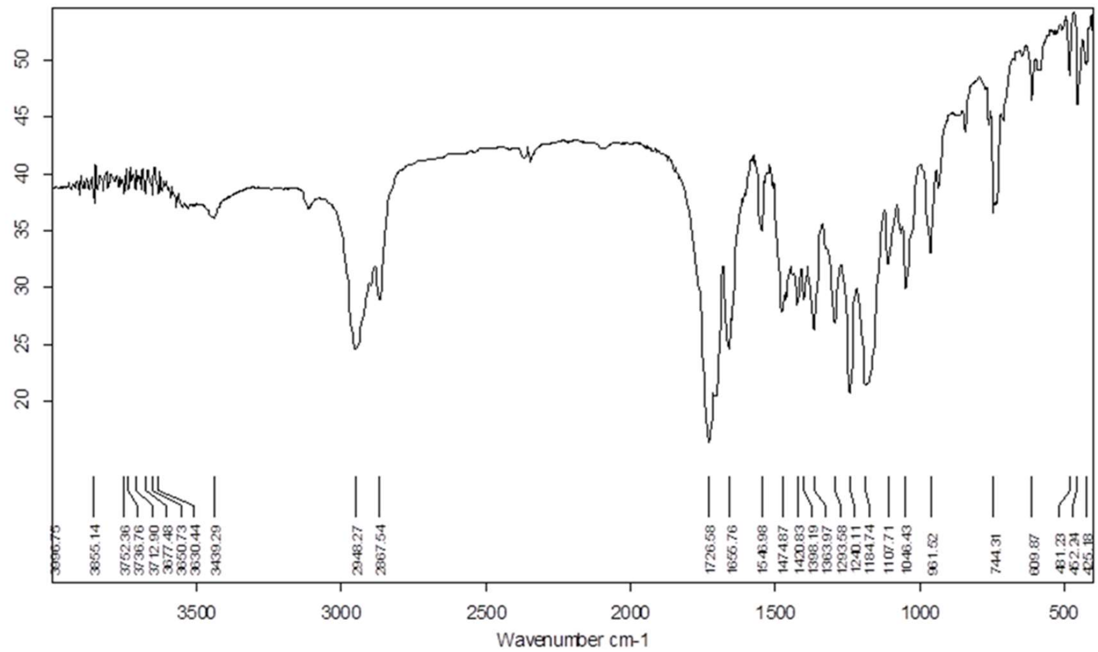
50
45
40
35
30
25
20
3996.75
3855.14
3752.36
3736.76
3712.90
3677.48
3650.73
3630.44
3439.29
2948.27
2867.54
1726.58
1655.76
1546.98
1474.87
1420.83
1398.19
1363.97
1293.58
1240.11
1184.74
1107.71
1046.43
961.52
744.31
609.87
481.23
452.24
425.18
3500
3000
2500
2000
1500
1000
500
Wavenumber cm-1

(**a**)

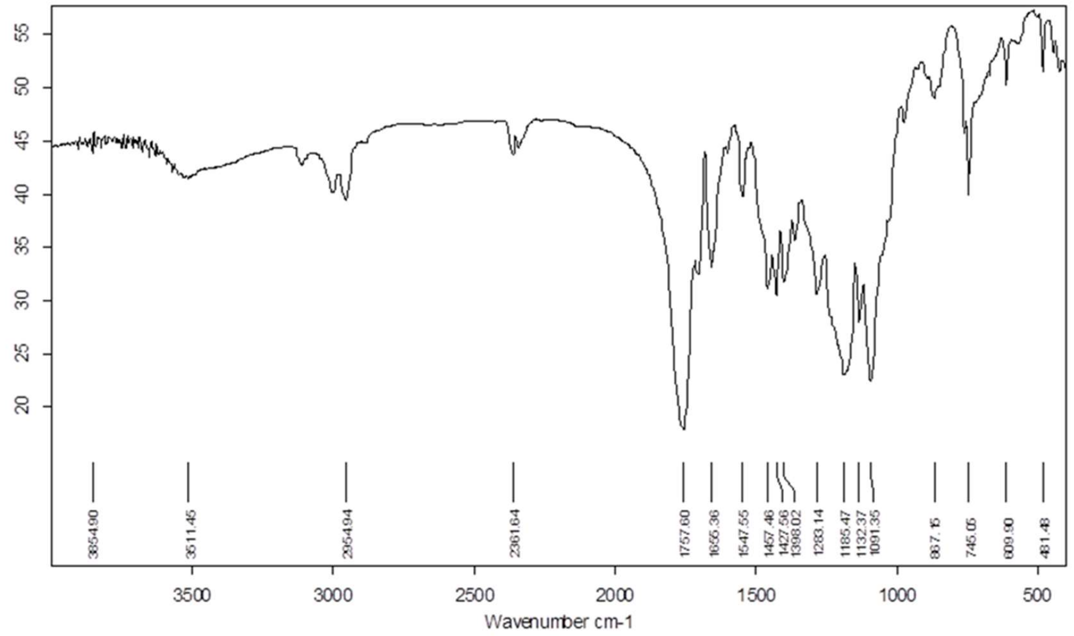
55
50
45
40
35
30
25
20
3854.90
3511.45
2954.94
2361.64
1757.60
1655.36
1547.55
1457.46
1427.56
1398.02
1283.14
1185.47
1132.37
1091.35
867.15
745.05
609.90
481.48
3500
3000
2500
2000
1500
1000
500
Wavenumber cm-1

(**b**)

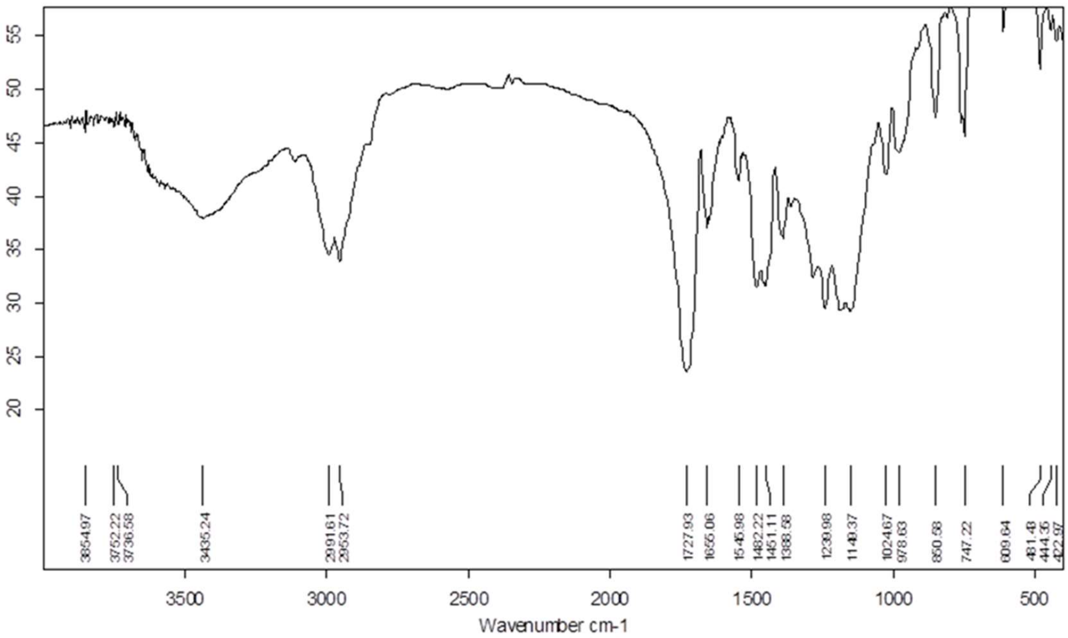

(**c**)

Figure S1. FTIR spectra of (**a**) PCL (**b**) PLGA and (**c**) ERL. Polymers were analyzed using a Bruker Vector 22 FTIR spectrophotometer. KBr discs with a 1% w/w sample loading were prepared by compression.
